# Cognitive behavioral therapy for bipolar disorder: patient-level participation barriers and ketogenic metabolic therapy as a candidate adjunct

**DOI:** 10.3389/fpsyg.2026.1782201

**Published:** 2026-06-15

**Authors:** Nicole Laurent

**Affiliations:** Independent Researcher, Vancouver, WA, United States

**Keywords:** bipolar disorder, cognitive behavior therapy, delivered psychotherapy dose, homework adherence, ketogenic diet, ketogenic metabolic therapy, metabolic psychiatry, psychotherapy engagement

## Abstract

This conceptual analysis synthesizes patient-level participation barriers described in the bipolar disorder CBT literature and considers ketogenic metabolic therapy (KMT) as a candidate adjunct within a delivery-focused framework. CBT is an evidence-based adjunctive psychotherapy for bipolar disorder, but its clinical value depends on whether patients initiate treatment, sustain session continuity, and complete between-session practice that supports skill use in daily life. In routine care, psychotherapy uptake remains limited, discontinuation can occur early, and patient-level barriers can constrain delivered dose. Early clinical evidence for KMT in bipolar disorder remains preliminary and does not support conclusions regarding disorder-level efficacy or comparative effectiveness. Case reports, retrospective analyses, and pilot trials describe KMT-associated change in domains that overlap with capacity for CBT participation, including mood stability, sleep, energy, anxiety, cognition, and functioning. To date, no studies have directly tested whether KMT improves CBT participation outcomes (initiation, continuity, or between-session practice) in bipolar disorder. This article presents a participation-capacity framework that evaluates KMT as an adjunct to guideline-consistent care and operationalizes research priorities for measuring participation, feasibility, and fidelity across psychotherapy dose, between-session practice, and verified ketosis, rather than symptom change alone. The model specifies sequential decision points for introducing KMT before CBT when participation capacity limits initiation or early continuity, or after participation barriers emerge. CBT is initiated or resumed when pre-specified participation-readiness indicators suggest that an adequate CBT dose can be delivered.

## 1 Introduction

Bipolar disorder (BD) is a recurrent disorder typically managed with pharmacotherapy, with contemporary treatment guidance emphasizing adjunctive psychosocial interventions as part of comprehensive care (Yatham et al., 2018; Keramatian et al., 2023). Within guidance from the Canadian Network for Mood and Anxiety Treatments (CANMAT) and the International Society for Bipolar Disorders (ISBD), structured psychosocial interventions are recommended as adjuncts to medication for relapse prevention and functional recovery (Oud et al., 2016; Yatham et al., 2018; Miklowitz et al., 2021; Keramatian et al., 2023). Psychoeducation is supported for relapse prevention during euthymia, and cognitive-behavioral therapy is positioned as an adjunctive option for acute bipolar depression and for maintenance treatment, with no recommendation for acute mania due to insufficient evidence in that phase (Chiang et al., 2017; Yatham et al., 2018; Keramatian et al., 2023).

Cognitive-behavioral therapy (CBT) for BD is a manualized psychotherapy that targets illness management skills for relapse prevention and depressive symptom management (Yatham et al., 2018). Evidence syntheses support mild-to-moderate effects when CBT is added to standard care, including reduced relapse, improved depressive symptoms, reduced mania severity, and improved psychosocial functioning in pooled randomized trial analyses (Chiang et al., 2017). Comparative syntheses of adjunctive manualized psychotherapies also support lower recurrence rates than control conditions, with CBT associated with stabilization of depressive symptoms at 12 months compared with treatment as usual (Miklowitz et al., 2021). Aggregate and individual patient data meta-analyses focused on bipolar depression similarly support psychological interventions as relevant to depressive symptom reduction, with findings most interpretable for CBT while also noting heterogeneity and limitations in evidence quality (Yilmaz et al., 2022, 2025b). Consistent with this evidence base, CBT is recommended as a second-line adjunctive treatment for acute bipolar depression and as a second-line option for maintenance in selected patients, with no recommendation for CBT in mania due to insufficient evidence (Yatham et al., 2018; Keramatian et al., 2023).

Despite guideline endorsement and evidence of benefit, psychotherapy remains underused in bipolar depression, and barriers to access and implementation mean that adequate delivery of evidence-based CBT cannot be assumed in routine care (Yatham et al., 2018; Chiang and Miklowitz, 2023). Attrition occurs even in structured CBT programs for bipolar disorder, with premature discontinuation limiting delivered dose (Jones et al., 2015; Docteur et al., 2018; Hautzinger and A2 BipoLife Consortium, 2024). In community settings, clinicians identify comorbid substance use disorders and treatment adherence difficulties as prominent barriers that interfere with optimal care delivery (Stein et al., 2015). Together, these findings support a delivery-focused framing in which CBT benefit depends on whether individuals can initiate and sustain sufficient participation for CBT to be delivered as intended (Docteur et al., 2018; Chiang and Miklowitz, 2023).

Ketogenic metabolic therapy (KMT) is a clinician-supervised ketogenic dietary intervention designed to induce nutritional ketosis and is increasingly discussed within metabolic psychiatry frameworks for serious mental illness, including mood disorders (Sethi and Ford, 2022; Rog et al., 2024). In BD, clinical literature has expanded from case-level reports (Yaroslavsky et al., 2002; Phelps et al., 2013; Saraga et al., 2020; Chmiel, 2022; Danan et al., 2022; Laurent, 2024; Schreel et al., 2025) to pilot studies supporting feasibility and acceptability and indicating the need for randomized controlled trials, including a BD-specific pilot and a pilot trial that enrolled participants with bipolar disorder alongside schizophrenia (Needham et al., 2023; Sethi et al., 2024). This conceptual analysis positions KMT as a candidate adjunct to CBT delivery in BD by framing whether KMT can reduce participation-limiting factors enough to increase CBT initiation, retention, and completion, with success defined in psychotherapy terms and KMT conceptualized as adjunctive rather than a replacement for established guideline-based BD treatments.

## 2 Positioning ketogenic metabolic therapy within CBT engagement and retention in bipolar disorder

Preclinical and mechanistic literature provides biological plausibility for investigating KMT in bipolar disorder. Animal and cellular work in mood-relevant models suggests that ketogenic states can alter pathways implicated in bipolar illness, including neuronal excitability, neuroinflammatory signaling, mitochondrial energy metabolism, and inhibitory to excitatory neurotransmitter balance, supporting a rationale for translational study without establishing clinical efficacy (Guan et al., 2020; Norwitz et al., 2020; Yu et al., 2023; Chrysafi et al., 2024; Freyberg et al., 2025). In parallel, theoretical frameworks in metabolic psychiatry propose bipolar-relevant models of dysregulated energy utilization and related ionic and circuit-level instability, positioning ketosis as a testable metabolic contrast within bipolar pathophysiology (Campbell and Campbell, 2024; Campbell et al., 2025). Against this backdrop, direct clinical evidence for KMT in bipolar disorder is drawn primarily from case reports, retrospective case series, and early feasibility and pilot trials. These reports describe KMT implementation and associated psychiatric outcomes in domains that may be relevant to CBT engagement, homework completion, and between-session follow-through, including mood stability, sleep, energy, anxiety, cognition, and functioning (Table 1).

**Table 1:** Patient-level participation barriers to CBT in bipolar disorder and bipolar KMT outcome domains reported as potentially relevant to these barriers.

Study/design	Participant(s)	Measurement and Timeline	Key findings relevant to CBT participation capacity
Yaroslavsky et al. (2002) case report	49-year-old woman, bipolar I, treatment resistant rapid cycling	1-month ketogenic diet trial. No standardized symptom scales were reported. Clinical outcome was reported only as no clinical improvement.	None. No participation capacity signal was reported because psychiatric change was not observed.
Phelps et al. (2013) case series; case A	69-year-old woman, bipolar II	No validated psychiatric symptom rating scales reported. Maintained ketogenic diet for approximately 2 years. Reported reduced agitation and no longer losing her temper; mood reported as calmer and stable.	Reduced agitation and improved behavioral control aligned with fewer mood driven disruptions to functioning.
Phelps et al. (2013) case series; case B	30-year-old woman, bipolar II	No validated psychiatric symptom rating scales reported. Self-reported calm mood and emotional stability reported in 2-3 days of ketogenic diet implementation: ability to maintain job and relationship at 3-year follow-up.	Rapid reported improvement in mood with sustained calm and stability aligned with fewer mood driven disruptions relevant to functioning.
Campbell and Campbell (2019), controlled analytic study of online reports	274 people with bipolar disorders in online forum reports. Ketogenic diet reports included 165 posts.	Duration was not consistently reported. Remission or significant improvement in mood stabilization was reported more often with ketogenic diet than comparison diets, 93/165 (56.4%) versus 14/94 (14.9%). Mood destabilization was reported in 8/165 (4.8%) ketogenic diet posts. Reported benefits attributed to ketogenic diet included improved mood stability 65%, fewer depressive episodes 41.2%, improved clarity of thought and speech 28.2%, increased energy 25.9%, reduced anxiety or panic attacks 20.0%, fewer episodes of mania 12.9%, improved sleep 8.2%, improved control of actions 8.2%, and improved memory 2.4%.	Reported remission or significant mood stabilization alongside improved clarity of thought and speech, increased energy, reduced anxiety or panic, improved sleep, improved control of actions, and improved memory may align with fewer symptoms and cognitive disruptions to functioning.
Saraga et al. (2020) case report	60-year-old woman, bipolar I	No validated psychiatric symptom rating scales reported. Decreased anxiety and maintenance of euthymia despite stressful life events reported. Patient narrative described depression lifting, more joy, improved ability to use psychotherapy tools, and reduced severity and duration of accelerated or irritable states.	Self-reported calmer mood with sustained stability over three years may align with fewer mood instability barriers to CBT participation continuity.
Chmiel (2022) case report	Male patient, ultra rapid cycling bipolar disorder	No validated psychiatric symptom rating scales reported. Described mood stabilization, increased energy, improved sleep, improved cognitive function and concentration, elimination of anxiety, shorter depressive periods, and no hypomania. Improvement reported occurring during the first year of the dietary intervention.	Improvements in energy, sleep, concentration, and anxiety may be aligned with reduced cognitive and motivational barriers that commonly limit between session practice and task follow through in CBT.
Danan et al. (2022) retrospective inpatient series	28 adult inpatients with severe, persistent mental illness, including 12 with bipolar disorder.	For the bipolar subgroup (n=12), the reported mood improvement was quantified through depressive symptoms and global severity scales. HAM-D 24.9 → 9.2, MADRS 29.9 → 11.8 (n=12), and CGI-S 4.8 → 2.0 (n=12), with changes presented as pre to post inpatient intervention.	Reduced depressive symptom severity and reduced global illness severity may align with fewer patient-level symptom burden barriers relevant to CBT participation continuity and between session practice.
Needham et al. (2023) single arm feasibility pilot in euthymic bipolar disorder with secondary clinical and EMA outcomes reported in Campbell et al., 2025	Euthymic bipolar disorder participants, 27 recruited; analysis done on 20 who completed the study.	Recruited to a 6 to 8 week modified ketogenic diet. Baseline to week 8 symptom scales collected, ALS 15 → 12.5 (n=18), YMRS 0 → 0 (n=20), BDI 8.5 → 9 (n=18), with scores remaining within euthymic range and no statistically significant change. Daily EMA collected for mood, energy, anxiety, impulsivity, and speed of thought, with ketone level associations analyzed in a subset with reliable EMA data (n=14). Process evaluation feedback noted perceived improvements in energy, mood, and self-control. Mood episodes were reported in some participants during or after the intervention, including hypomania and depression episodes with resolution described.	EMA and process evaluation suggested higher mood and energy with lower anxiety and impulsivity during the intervention, which may align with fewer low energy, anxiety, and self regulation barriers to between session follow through, while mood episodes were reported in some participants.
Sethi et al. (2024) (single-arm pilot trial)	16 adults with bipolar disorders within a 21-participant pilot cohort.	Bipolar subgroup CGI improvement greater than 1 point in 69% with CMF recovered state increasing from 38% to 81%. Full cohort reported CGI severity of mental illness (baseline 3.6 ± 1.0; change −1.3 ± 1.3 at 4 months; full cohort); GAF (baseline mean 63.8 ± 8.0; 17% improvement at 4 months; full cohort); MANSA quality of life (baseline mean 4.3 ± 1.0; change +0.6 ± 0.7 at 4 months; full cohort); PSQI sleep disturbance (baseline mean 7.9 ± 3.7; change −1.7 ± 2.8 at 4 months; full cohort)	Concurrent improvements in depressive symptoms, anxiety, sleep, functioning, and global severity may align with patient-level barriers to improve CBT initiation, continuity, and between session follow through.
Laurent (2024) retrospective case study	53-year-old woman, bipolar II, treatment-resistant	Mood assessments collected at baseline, 1-month, 4-month, and 5-month intervals over a 21-week period. GAD-7 8 → 1, PCL-5 38 → 8 (Criterion D 16 → 3 and Criterion E 14 → 2), DASS-42 Depression 16 → 4, Anxiety 17 → 9, Stress 15 → 2. Qualitative analysis described improved emotional well-being, enhanced quality of life, improved daily functioning, and increased autonomy in treatment decision-making.	Reduced depressive and anxiety related distress and reduced cognition and mood and arousal symptoms may align with fewer patient-level emotional distress and concentration barriers to CBT participation continuity and between-session follow-through.
Rigby et al. (2025) qualitative process evaluation	15 participants with bipolar disorder interviewed 1 to 2 months post-intervention and 4 research clinicians from Needham et al. (2023).	Thematic analysis reported five themes, with Theme 2, Challenging but potentially transformational, captured that initiation and maintenance could be difficult while many participants perceived physical and psychological benefits, including mood stability and enhanced ability to focus.	Perceived mood stability and enhanced ability to focus aligned with fewer patient-level barriers such as mood instability and cognitive burden.
Schreel et al. (2025), outpatient case series, Case 1	39-year-old single man with bipolar disorder II	12 week intervention. GAF 60 → 100. CGI BD S 5 → 2. PHQ 9 13 → 1. BDI II 20 → 3. HAM D 14 → 2. MADRS 22 → 1. Narrative described improved emotional resilience, energy, focus, willpower, and moving from part-time to full-time work.	Large improvements in depression severity and functioning may align with reduced patient-level barriers to CBT initiation, session continuity, and between-session follow-through.
Schreel et al. (2025), outpatient case series, case 2	50-year-old woman with rapid-cycling bipolar disorder II	12 week intervention. GAF 50 → 80. CGI BD S 5 → 2. PHQ 9 18 → 3. BDI II 42 → 7. HAM D 21 → 5. MADRS 26 → 7. Reported “remarkable improvements in all psychiatric assessments,” without additional patient-reported mood, cognition, or functioning descriptions beyond the scale changes and GAF.	Reduced depressive severity and improved functioning aligned with improved capacity for session continuity and completion of between session assignments that require planning and sustained effort.
Schreel et al. (2025), outpatient case series, case 3	46-year-old man with bipolar disorder II	12-week intervention. GAF 95 → 100. CGI BD S 4 → 2. PHQ 9 6 → 2. BDI II 6 → 2. HAM D 5 → 3. MADRS 7 → 1. Reported high beginning baseline functioning and low symptom burden with small pre to post changes on the depression and severity measures. Reported increases energy and emotional resiliency with reduced irritability, described as “life-changing” and corroborated by friends and family	Experiences of improved energy and emotional resiliency may align with fewer fatigue/low-energy and affective reactivity patient-level barriers that can disrupt CBT continuity and between-session follow-through.
Schreel et al. (2025), outpatient case series, case 4	47-year-old woman with bipolar disorder II	12-week intervention. GAF 60 → 100. CGI BD S 3 → 2. PHQ 9 9 → 0. BDI II 13 → 1. HAM D 9 → 0. MADRS 8 → 0. Reports did not include additional patient-described mood, cognition, or functioning changes beyond the scale and GAF improvements.	Reduced depressive severity and improved functioning may align with fewer mood related patient-level barriers to CBT initiation, session continuity, and between-session follow-through.
Schreel et al. (2025), outpatient case series, case 5	34-year-old woman with bipolar disorder I	12-week intervention. GAF 60 → 100. CGI-BD-S 4 → 1. PHQ-9 3 → 2. BDI-II 8 → 3. HAM-D 7 → 0. MADRS 9 → 1. Reported improvements across all psychiatric assessments, without additional patient-reported mood, cognition, or functioning detail beyond GAF.	Improved global functioning and reduced overall illness severity may align with fewer patient-level barriers of symptom burden relevant to CBT initiation, session continuity, and between-session follow-through.
Schreel et al. (2025), outpatient case series, case 6	29-year-old man with bipolar disorder I	12-week intervention. GAF 80 → 100. CGI-BD-S 3 → 4. PHQ-9 1 → 2. BDI-II 9 → 5. HAM-D 2 → 3. MADRS 3 → 2. Mixed outcomes were reported, with improved global functioning alongside limited improvement in depression ratings and worsening on overall bipolar severity.	Mixed response did not support a clear reduction in patient-level barriers and highlighted heterogeneity and the need for individualized sequencing decisions rather than assuming CBT readiness improves.
Schreel et al. (2025), outpatient case series, case 7	50-year-old man with bipolar disorder I	12-week intervention. Reported as non-responder. GAF 60 → 70. CGI-BD-S remained 4. PHQ-9 8 → 9. BDI-II 8 → 7. HAM-D 10 → 10. MADRS 6 → 12.	Reports of non-responder status are relevant to research questions to better understand who may benefit from KMT as an adjunct to reduce patient-level barriers to CBT and who may not.

Summary of published bipolar disorder reports and early studies of ketogenic metabolic therapy, organized by study design, participant characteristics, measurement and timeline, and outcome domains mapped to patient-level barriers that commonly limit cognitive behavioral therapy participation and between-session follow-through, including mood stability, energy, anxiety, cognition, behavioral control, and functioning. The table is intended to support a delivery-focused interpretation of reported KMT-associated change, while noting heterogeneity in design, variable use of validated scales, and mixed responder patterns across reports. Acronyms: ALS, Affective Lability Scale, BPRS, Brief Psychiatric Rating Scale; CBTp, cognitive behavioral therapy for psychosis; CGI-S, Clinical Global Impressions–Severity; CMF, Clinical Mood Form; DASS-42, Depression Anxiety Stress Scales (42-item); GAD-7, Generalized Anxiety Disorder−7; GAF, Global Assessment of Functioning; HAM-D, Hamilton Depression Rating Scale; KMT, ketogenic metabolic therapy; MADRS, Montgomery–Åsberg Depression Rating Scale; MANSA, Manchester Short Assessment of Quality of Life; PANSS, Positive and Negative Syndrome Scale (*P* = Positive, *N* = Negative, *G* = General Psychopathology subscales); PCL-5, PTSD Checklist for DSM-5; PHQ-9, Patient Health Questionnaire−9; PSQI, Pittsburgh Sleep Quality Index; SD, standard deviation. EMA, Ecological momentary assessment.

An inpatient case report described an attempted ketogenic diet intervention in a treatment-resistant patient with rapid-cycling bipolar I disorder. The report described **4** weeks of diet implementation during hospitalization, with adherence rated as very good, and it noted no clinical improvement over the intervention period. The authors indicated that the intended ketogenic intervention was not successfully established during the admission and ketosis was not confirmed, limiting outcome interpretation (Yaroslavsky et al., 2002).

In a two-case report of bipolar II disorder, the report described a 69-year-old woman who had achieved partial benefit on medication but continued to experience residual depressive burden and prominent irritability, with dose escalation limited by side effects. After initiating a ketogenic diet, she reported improved mood stability and a marked reduction in agitation and “hair-trigger” reactivity, describing a sustained shift in how she responded emotionally to stressors and day-to-day frustrations, with benefits centered on steadier mood and improved behavioral control in daily life (Phelps et al., 2013). In the same report, a 30-year-old woman with bipolar II disorder with onset in adolescence reported adverse reactions to prior antidepressant trials, including increased suicidality, and later achieved sufficient stability with medication to sustain employment and a stable relationship. She initially adopted a ketogenic diet during flares of gastrointestinal symptoms and noticed that her mood felt calmer while in ketosis. Later, when she discontinued medication in preparation for pregnancy, she returned to the ketogenic diet, reported that mood symptoms resolved within days of achieving ketosis, and described sustained stability over multiple years during continued dietary implementation (Phelps et al., 2013).

A forum-based observational study analyzed free-text posts from individuals who self-reported bipolar disorder and described mood effects of dietary interventions. Reports of significant mood stabilization or symptom remission over time were substantially more common among those describing a ketogenic diet. In addition to mood stability, the analysis identified posts reporting fewer depressive episodes, fewer manic episodes, improved clarity of thought and speech, increased energy, reduced anxiety or panic attacks, and improved sleep. The authors emphasized that these findings were derived from self-reported public forum posts and should be interpreted cautiously (Campbell and Campbell, 2019).

A single case report described ketogenic diet use in bipolar I disorder that was self-initiated at home. The report described a 60-year-old woman with bipolar I disorder and a long history of mixed episodes with psychotic symptoms, who began a ketogenic diet after prior intensive psychotherapy. During follow-up, the clinical narrative reported decreased anxiety and maintenance of euthymia despite markedly stressful life events. The patient reported improved capacity to use her psychotherapy tools when difficulties arose. Brief mood dysregulations were described as shorter and less impairing than prior patterns (Saraga et al., 2020).

A subsequent case report described a male patient with bipolar disorder and ultra-rapid cycling who adopted a ketogenic dietary approach after persistent mood instability despite prior treatment. During multi-year follow-up, the report described the absence of hypomanic episodes and substantial improvement in depressive symptoms, with depressive periods becoming shorter and less severe. The narrative also noted improved sleep quality and improved cognitive functioning, including improved concentration (Chmiel, 2022).

A retrospective inpatient analysis examined adults with severe, persistent mental illness treated in a psychiatric hospital setting who received a ketogenic diet as an adjunct to standard inpatient care. **13** of the **31** patients were diagnosed with bipolar II disorder. In this subgroup, clinician-rated depressive symptom burden and global illness severity improved from pre- to post-intervention, consistent with reduced depressive symptoms and improved overall clinical status during hospitalization, within a structured clinical setting in which symptom change was tracked using established rating scales (Danan et al., 2022).

A single-arm prospective pilot study evaluated the feasibility, safety, and preliminary clinical signals of a 6 to 8 week modified ketogenic diet intervention in clinically euthymic adults with bipolar disorder delivered with structured dietetic support. Feasibility outcomes and process evaluation were reported in the initial publication, including daily ecological momentary assessment of anxiety, mood, energy, impulsivity, and speed of thought and participant feedback describing perceived improvements in mood, energy, and self-control during the intervention (Needham et al., 2023). A subsequent publication from the same cohort reported secondary clinical and daily rating findings, including associations between higher ketone levels and better day to day mood and energy with lower anxiety and impulsivity, and noted that seven of 20 completers declined the planned diet cessation period and opted to continue the ketogenic diet (Campbell et al., 2025). In a further publication from the same intervention, a mixed methods process evaluation examined feasibility, acceptability, and implementation using semi-structured post-intervention interviews with participants and intervention clinicians alongside descriptive analysis of fidelity checklists capturing behavior change components. Participants reported perceived psychological benefits, including greater mood stability, improved ability to focus, and improved capacity to tolerate stressors (P. Rigby et al., 2025).

A retrospective single-case study examined a woman with bipolar II disorder and longstanding treatment-resistant depressive symptoms. At the time of diet implementation, psychiatric intervention included weekly ketamine treatments that provided only short-lived relief, with benefits lasting **1** to **3** days before symptoms returned. KMT was delivered with clinician supervision and structured education. Over a 21-week period, the report described normalization of depressive and anxiety symptom scores alongside improved daily functioning and quality of life, and the case narrative and qualitative analysis emphasized greater energy, reduced fatigue, and improved capacity to manage everyday demands (Laurent, 2024).

A 4-month single-arm outpatient pilot trial enrolled adults with bipolar disorder or schizophrenia who continued their usual psychiatric treatment while receiving structured ketogenic diet support and monitoring. Among those who completed the study, overall clinical severity ratings improved over the intervention period, alongside improved sleep quality, improved life satisfaction, and improved global functioning. In subgroup analyses, bipolar participants showed improvement on overall severity ratings with a higher proportion rated as recovered on mood monitoring by study end (Sethi et al., 2024).

An outpatient case series reported seven individuals with bipolar disorder (four with bipolar II and **three** with bipolar I) who completed a 3-month ketogenic intervention while psychiatric treatment remained unchanged. Across the seven cases, the authors described improvement across all psychiatric assessments in **5** participants, while **one** case showed mixed changes and was not considered an unequivocal responder and **one** was characterized as a non-respondent. Global bipolar illness severity improved in five, was unchanged in one, and worsened in one. Depressive symptom measures generally improved across the series, alongside patient reports that included increased energy and emotional resilience or steadier mood in **two** cases and improved mental focus with better occupational functioning in **one** case (Schreel et al., 2025).

Across these case reports, retrospective series, and early pilot studies, the evidence base for KMT in bipolar disorder remains inconclusive and does not yet support broad conclusions regarding disorder level efficacy or comparative effectiveness (Rog et al., 2024). This early literature does however, describe consistent patient experiences across most case reports and trials, including improved mood stability, sleep regulation, reduced anxiety, improved cognitive function, increased energy, and improved day-to-day functioning, including among individuals with complex comorbidity and treatment-resistant illness (Phelps et al., 2013; Saraga et al., 2020; Chmiel, 2022; Danan et al., 2022; Needham et al., 2023; Laurent, 2024; Sethi et al., 2024; Schreel et al., 2025). These findings do not by themselves establish improved participation capacity; rather, they identify domains that are clinically relevant to patient-level factors reported to shape engagement, retention, and delivered dose of effective psychotherapy treatment.

## 3 Patient-level barriers to CBT engagement, homework and follow-through in bipolar disorder

A delivery-focused framing requires specifying what counts as participation in CBT for bipolar disorder. Participation includes initiation of treatment, continuity of attendance across the intended course, and completion of between-session tasks that extend the active ingredients of CBT into daily life (Miklowitz et al., 2021). CBT protocols for bipolar disorder commonly rely on repeated practice of monitoring, planned behavior change, and relapse prevention skills (ÖZDEL et al., 2021). Clinical benefit depends on a sustained dose of treatment delivered over time rather than isolated sessions (Chiang and Miklowitz, 2023). Evidence syntheses support that adjunctive CBT produces benefit in bipolar disorder when delivered as intended, while also underscoring that psychotherapy effects are contingent on sufficient engagement and retention (Oud et al., 2016; Chiang et al., 2017; Miklowitz et al., 2021). Psychotherapy participation in bipolar depression is constrained by underuse, limited uptake, and premature discontinuation in routine care, and adequate CBT delivery cannot be assumed outside research settings (Docteur et al., 2018; Miklowitz et al., 2021; Chiang and Miklowitz, 2023). Even in structured CBT trials for bipolar disorder, some participants do not complete the full intervention (Chiang et al., 2017), and early discontinuation can limit the delivered dose (Jones et al., 2015; Docteur et al., 2018).

Patient-level barriers to CBT engagement refer to clinical, cognitive, motivational, and social factors that interfere with initiation, session continuity, and completion of between-session practice (Stein et al., 2015; Hanssen et al., 2020; Chiang and Miklowitz, 2023). In bipolar disorder, these barriers commonly include depressive symptoms associated with low energy and reduced concentration (Hanssen et al., 2020). They also include hypomanic or manic symptoms (Hanssen et al., 2020) that disrupt continuity, limited insight and inaccurate self-appraisal (Grover et al., 2023; Wels et al., 2023), comorbid substance use and adherence difficulties (Stein et al., 2015), and limited social stability and support that undermine follow-through (Veseth et al., 2017).

Cognitive burden is another participation limiting factor. CBT depends on sustained attention, working memory, and the ability to translate session content into plans that are implemented between sessions. Qualitative findings describe depression related reductions in concentration and energy that interfere with participation and between-session practice (Hanssen et al., 2020). A further complication is that self-appraisal of cognitive performance is inaccurate for some individuals with bipolar disorder (Wels et al., 2023). Evidence describes state linked patterns of overestimation and underestimation that undermine realistic planning and calibration of task demands (Wels et al., 2023). Poorer insight is associated with greater residual symptoms, cognitive impairment, and disability with participation constraints related to cognition and insight persisting outside acute episodes (Grover et al., 2023).

Between-session homework demands introduce a distinct burden. They require effort when symptoms, motivation, and self-regulation are already strained. CBT for bipolar disorder commonly includes self-monitoring and structured tasks designed to build skills. Patients struggle to complete these tasks during depression due to low motivation and negative expectations. They also struggle during elevated states due to difficulty disengaging from reinforcing activities (ÖZDEL et al., 2021). Qualitative evidence indicates that homework volume feels excessive for some participants and triggers self-imposed pressure that reduces continuation of practice (Hanssen et al., 2020).

Insight and therapeutic alliance function as participation mechanisms. They shape collaboration, adherence, and willingness to sustain treatment (Strauss and Johnson, 2006; Grover et al., 2023). Poorer insight is associated with greater illness burden and functional impairment reducing consistency of engagement and follow-through (Grover et al., 2023). Treatment alliance is associated with clinically relevant outcomes in bipolar disorder (Strauss and Johnson, 2006) Engagement and continuity depend on the therapeutic relationship as well as symptom severity (Strauss and Johnson, 2006). In routine care, psychotherapy underuse also reflects patient reticence and practical adherence difficulties with engagement barriers occurring before homework failure becomes observable (Chiang and Miklowitz, 2023).

Comorbidity and adherence problems are prominent barriers in community settings where CBT is delivered. In routine care, these barriers commonly involve co-occurring conditions and adherence problems that reduce continuity of participation. Clinicians identify comorbid substance use disorders and adherence difficulties as barriers that interfere with psychosocial care delivery for bipolar disorder (Stein et al., 2015). Guideline summaries also emphasize the clinical importance of comorbidity and adherence constraints in bipolar disorder care (Yatham et al., 2018; Keramatian et al., 2023). Participation barriers often occur within complex clinical profiles rather than in narrowly defined trial samples (Jones et al., 2015; Docteur et al., 2018; Keramatian et al., 2023).

Social context influences participation capacity through stability and practical support. It also shapes whether patients have structured support that helps them sustain treatment routines (Veseth et al., 2017; Yatham et al., 2018; Keramatian et al., 2023). In bipolar disorder, therapists describe the social world as central to recovery stability, with instability undermining continuity and follow through, and treatment alliance is linked with social support, indicating that participation barriers are embedded in the patient's broader context rather than solely in individual motivation (Strauss and Johnson, 2006; Veseth et al., 2017).

These findings support a delivery-focused problem statement. CBT is effective in bipolar disorder (Chiang et al., 2017). However, patient-level barriers often limit initiation, retention, and between-session practice (Docteur et al., 2018; ÖZDEL et al., 2021; Chiang and Miklowitz, 2023). This reduces delivered dose and constrains real-world benefit (Docteur et al., 2018; Chiang and Miklowitz, 2023). This framing supports evaluating adjunct strategies based on whether they reduce participation burdens enough to increase engagement and between-session follow-through. Success is defined in psychotherapy terms rather than solely in symptom change (Chiang and Miklowitz, 2023).

## 4 Ketogenic metabolic therapy as a candidate adjunct for participation capacity in bipolar disorder

Adjunct psychotherapy trials in bipolar disorder prioritize clinical outcomes, while participation processes are less consistently characterized (Szentagotai and David, 2010; Oud et al., 2016). In systematic syntheses, study retention is commonly used as a proxy for treatment acceptability, whereas more granular indicators that clarify delivered psychotherapy dose and feasibility, including session attendance frequency, medication adherence, and patient ratings of helpfulness, are reported less often (Gaudiano et al., 2008; Miklowitz et al., 2021).

A participation-capacity model treats these measurement gaps as clinically relevant because CBT benefit depends on sufficient delivered dose and between-session practice (Docteur et al., 2018; Miklowitz et al., 2021; ÖZDEL et al., 2021). A candidate adjunct is evaluated based on whether it improves initiation (Chiang and Miklowitz, 2023), session continuity (Docteur et al., 2018; Miklowitz et al., 2021), and between-session follow-through (ÖZDEL et al., 2021), with success defined in psychotherapy terms rather than symptom change as the sole endpoint (Chiang and Miklowitz, 2023).

**Figure 1:**
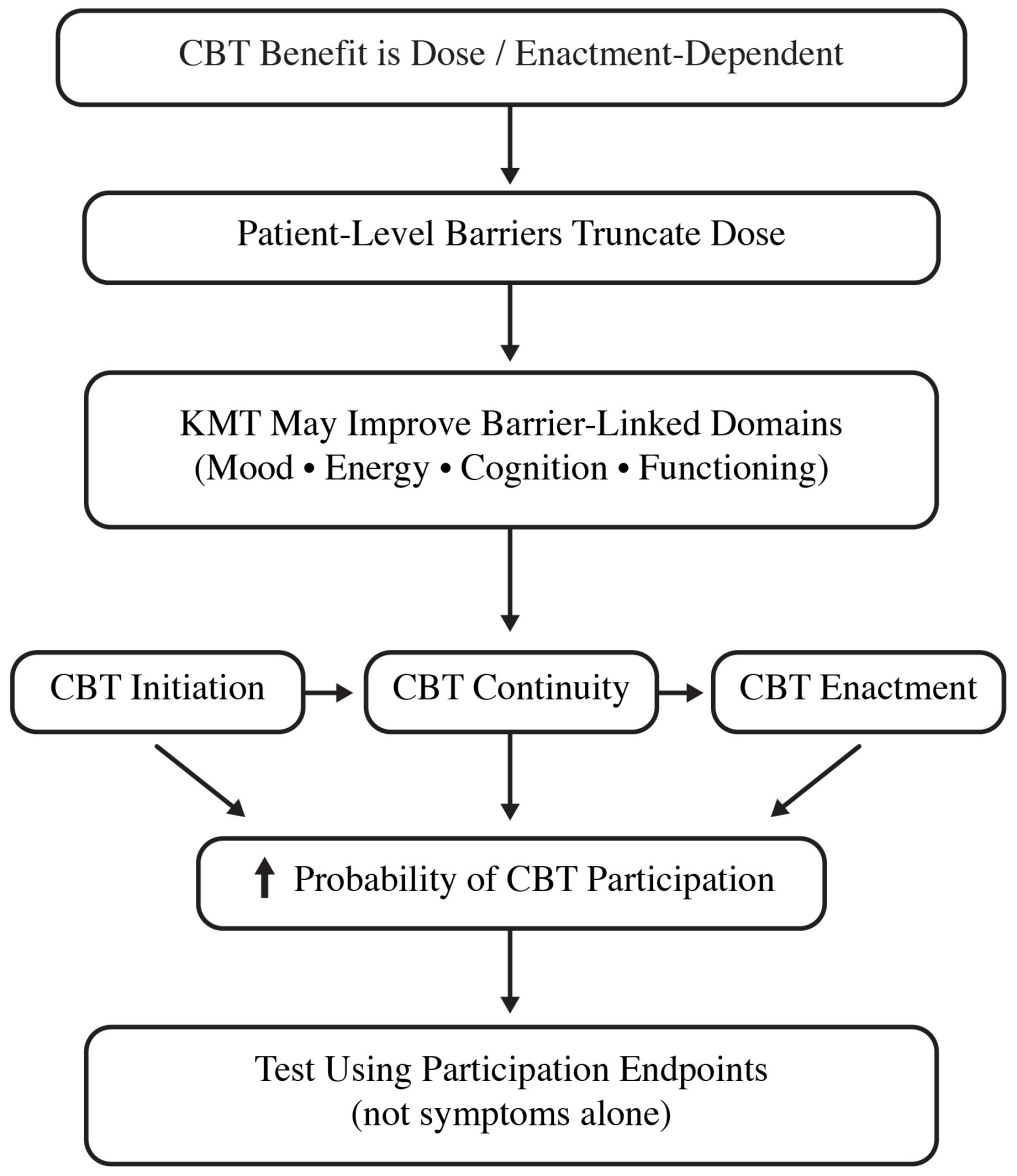
KMT participation-capacity framework for patient-level barriers to CBT delivery in bipolar disorder (BD). Conceptual pathway in which KMT-associated change in patient-level barrier-linked domains (mood, energy, cognition, functioning) may increase successful delivery of an adequate CBT dose via improved initiation, continuity, and enactment. The conceptual framework is evaluated using CBT participation endpoints rather than symptoms alone.

Figure 1 presents the paper's conceptual pathway linking dose and enactment dependent CBT benefit with patient-level barriers that can truncate delivered dose, and positioning KMT related domains of functioning as candidate targets that may increase CBT initiation, CBT continuity, and CBT enactment. Within this framework, KMT is considered a candidate adjunct only to the extent that it is associated with change in domains that overlap with participation capacity. Table 2 summarizes patient-level CBT engagement barriers and preliminary KMT findings relevant to psychotherapy participation in bipolar disorder.

**Table 2:** Patient-level participation barriers and KMT findings relevant to CBT delivery in bipolar disorder.

Research	Patient-level barriers to adequate CBT dose and engagement	Preliminary reports of KMT improvements in these identified barriers	Where KMT may be introduced as a clinical adjunct
Chiang and Miklowitz (2023) narrative review	Skepticism of psychotherapy benefit and reticence to engage in treatment were described as barriers to uptake. Depressive symptom burden, including subsyndromal depressive symptoms linked to psychosocial disability and reduced community functioning, may constrain CBT initiation and continuity (Chiang and Miklowitz, 2023)	Depressive symptoms (Danan et al., 2022; Laurent, 2024; Sethi et al., 2024), Functioning (Phelps et al., 2013; Sethi et al., 2024; Laurent, 2024), Global illness severity (Danan et al., 2022; Sethi et al., 2024).	When CBT is indicated for bipolar depression or maintenance, but psychotherapy is not initiated due to depressive symptom burden, reticence, or skepticism about benefit, KMT may be considered alongside standard care to support initiation and early continuity.
Docteur et al. (2018) observational cohort study	20-session outpatient CBT program for bipolar I disorder; all dropouts occurred before session 5. Dropouts had higher baseline Mania Rating Scale scores despite low mood symptoms overall, consistent with subsyndromal activation as a potential barrier to early attendance continuity and delivered dose.	Mood stability (Phelps et al., 2013; Campbell and Campbell, 2019; Saraga et al., 2020; Chmiel, 2022; Needham et al., 2023; Rigby et al., 2025), Lower impulsivity (Needham et al., 2023; Campbell et al., 2025), Improved control of actions (Campbell and Campbell, 2019), Reduced agitation or irritability (Phelps et al., 2013)	When early attendance continuity is disrupted by subsyndromal activation, KMT may be considered alongside standard care to support mood stabilization and self-regulation sufficient for completion of a minimally adequate CBT course.
Grover et al. (2023) Observational cross-sectional study	Poor insight was associated with higher residual depressive and manic symptoms, greater cognitive impairment, and higher disability in bipolar disorder. This pattern may constrain CBT collaboration and follow-through. functioning, may constrain CBT initiation and continuity	Cognitive function and concentration (Chmiel, 2022), Clarity of thought and speech (Campbell and Campbell, 2019), Ability to focus (Rigby et al., 2025), Memory (Campbell and Campbell, 2019), Reduced cognitive burden (Laurent, 2024), Global functioning (Sethi et al., 2024), Global illness severity (Danan et al., 2022; Sethi et al., 2024), Functioning and quality of life (Laurent, 2024)	When insight limitations and cognitive impairment constrain CBT collaboration and between-session follow-through, KMT may be considered alongside standard care with the objective of improving cognitive and global functioning to support collaboration and follow-through.
Hanssen et al. (2020) qualitative study, grounded theory	Depressive symptoms were described as a barrier to participation due to low energy, poor concentration, and reduced motivation. Homework demands were commonly experienced as too much, with self-imposed pressure reducing continuation of practice, and hypomanic or manic symptoms were described as interfering with participation and concentration.	Energy (Campbell and Campbell, 2019; Needham et al., 2023), Cognitive function and concentration (Chmiel, 2022), Clarity of thought and speech (Campbell and Campbell, 2019), Ability to focus (Rigby et al., 2025), Mood stability (Phelps et al., 2013; Campbell and Campbell, 2019; Chmiel, 2022), Fewer episodes of mania (Campbell and Campbell, 2019; Chmiel, 2022), Improved control of actions (Campbell and Campbell, 2019), Lower impulsivity and improved self-control (Needham et al., 2023; Campbell et al., 2025)	When low energy, poor concentration, reduced motivation, and mood instability undermine between-session practice, adjunct KMT may be considered alongside standard care to support energy, concentration, and mood stability sufficient for completion of a minimally adequate CBT course.
ÖZDEL et al. (2021) narrative review	Loss of motivation and negative expectations were described as reasons mood monitoring is not completed during depressive episodes, and difficulty disengaging from reinforcing behaviors was described as a barrier during hypomanic or manic episodes.	Mood stability (Phelps et al., 2013; Campbell and Campbell, 2019; Chmiel, 2022; Rigby et al., 2025), Reduced depressive symptoms (Danan et al., 2022; Laurent, 2024; Sethi et al., 2024), Lower impulsivity and improved self-control (Needham et al., 2023; Campbell et al., 2025), Improved control of actions (Campbell and Campbell, 2019)	When mood monitoring and between-session follow-through fail during depressive or elevated states, KMT may be considered alongside standard care to support mood stability and self-regulation that sustain monitoring and follow-through.
Stein et al. (2015) observational cross-sectional survey study	Clinicians reported substance use problems interfering with treatment (49%) and substance use being more pressing than bipolar symptoms (44%). Many also endorsed poor adherence to bipolar disorder treatment (56%). Less commonly endorsed barriers were poor reimbursement or limited benefits (15%), short sessions (9%), and inadequate follow up (7%).	Mood stability (Phelps et al., 2013; Campbell and Campbell, 2019; Saraga et al., 2020; Chmiel, 2022; Needham et al., 2023; Rigby et al., 2025), Self-control and lower impulsivity (Needham et al., 2023; Campbell et al., 2025), Improved control of actions (Campbell and Campbell, 2019), Depressive symptoms (Danan et al., 2022; Laurent, 2024; Sethi et al., 2024), Energy (Campbell and Campbell, 2019; Needham et al., 2023), Cognitive function and concentration (Chmiel, 2022), Clarity of thought and speech (Campbell and Campbell, 2019), Ability to focus (Rigby et al., 2025)	When substance use, adherence difficulties, and service constraints limit CBT continuity, adjunct KMT may be considered alongside standard care. Reported KMT improvements in mood stability, self-control, and cognitive functioning may align with improved participation capacity.
Strauss and Johnson (2006) observational prospective longitudinal study	Alliance strength decreased as depressive symptoms increased within individuals over time, indicating that depressive symptom burden may erode the alliance needed to sustain collaborative psychotherapy work.	Depressive symptoms (Danan et al., 2022; Laurent, 2024; Sethi et al., 2024), Fewer episodes of mania (Campbell and Campbell, 2019; Chmiel, 2022), Mood stability (Phelps et al., 2013; Campbell and Campbell, 2019; Saraga et al., 2020; Chmiel, 2022; Needham et al., 2023; Rigby et al., 2025)	When depressive symptom burden erodes therapeutic alliance and disrupts continuity, KMT may be considered alongside standard care to support mood stability sufficient for sustained alliance and completion of a minimally adequate CBT course.
Wels et al. (2023) scoping review	Cognitive insight and introspective accuracy were described as impaired in bipolar disorder, with overestimation of cognitive performance common and underestimation occurring during depressive episodes. State-linked overestimation and underestimation related to cognitive performance and impaired functioning may disrupt realistic self-appraisal needed for task calibration and follow-through in CBT.	Cognitive function and concentration (Chmiel, 2022), Clarity of thought and speech (Campbell and Campbell, 2019), Ability to focus (Rigby et al., 2025), Memory (Campbell and Campbell, 2019), Reduced cognitive burden (Laurent, 2024)	When cognitive burden and reduced concentration undermine task calibration and between-session follow-through, adjunct KMT may be considered with the objective of improving cognitive function and concentration sufficiently to support task calibration and sustained follow-through.

This table summarizes and includes bipolar disorder psychotherapy and implementation references that describe patient-level barriers to initiating CBT, sustaining session continuity, or completing between-session practice, and it excludes efficacy-focused meta-analyses and guideline summaries that do not report patient-level participation barriers. It is not intended to provide an exhaustive inventory of participation barriers found in the research literature or to attribute causal effects to KMT. Acronyms: BD, bipolar disorder; CBT, cognitive behavioral therapy; KMT, ketogenic metabolic therapy.

This positioning keeps the adjunct question narrow and testable within guideline-consistent care. CBT is not recommended in acute mania (Yatham et al., 2018; Keramatian et al., 2023), and sufficient participation capacity is a prerequisite for collaborative CBT work and between-session practice (Yatham et al., 2018; Chiang and Miklowitz, 2023). This framing supports clinical implementation models that use psychotherapy participation endpoints to specify when an adjunct question is reached and to determine whether KMT functions as a meaningful adjunct in a given case (Miklowitz et al., 2021; Chiang and Miklowitz, 2023).

## 5 Clinical implementation of ketogenic metabolic therapy as an adjunct to cognitive behavioral therapy in bipolar disorder

Clinical implementation of KMT as an adjunct to CBT is most coherent when the goal is defined in psychotherapy terms. The operational objective is improved capacity to initiate CBT, sustain session continuity across an intended course, and complete between-session monitoring and skill practice, rather than symptom change as the sole indicator of success. This positioning remains bounded by guideline framing in which CBT is an adjunctive option for bipolar depression and maintenance, with no recommendation for acute mania due to insufficient evidence in that phase (Yatham et al., 2018; Chiang and Miklowitz, 2023; Keramatian et al., 2023).

**Figure 2:**
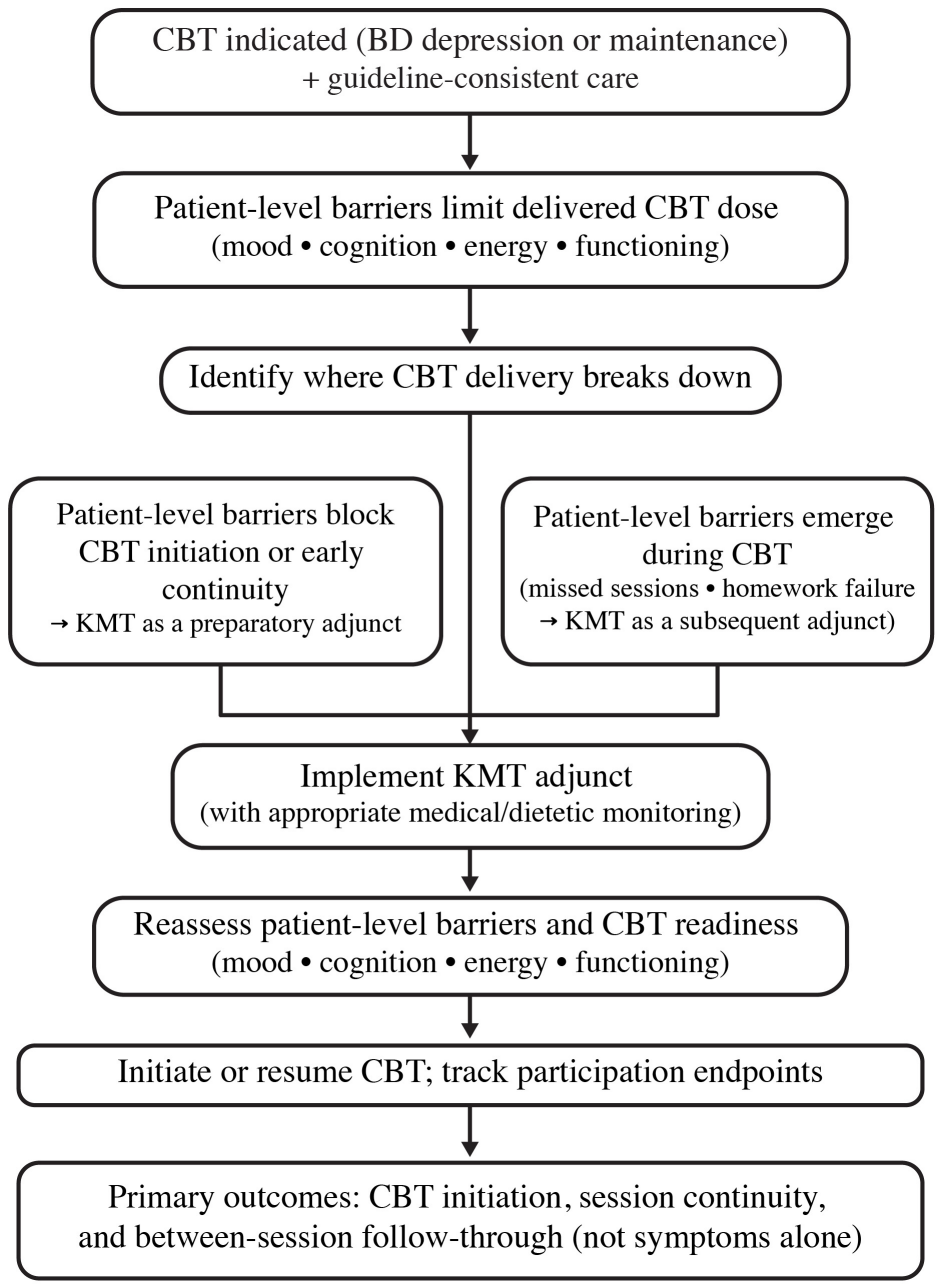
KMT participation-capacity sequencing model for patient-level barriers limiting delivered CBT dose in bipolar disorder (BD). Conceptual sequencing model for introducing KMT as a preparatory or subsequent adjunct to CBT in bipolar disorder when patient-level barriers limit delivered CBT dose. The model distinguishes pre-CBT participation-readiness indicators used to guide initiation or resumption of CBT from CBT participation endpoints used after CBT begins, including initiation, session continuity, and between-session follow-through.

A participation-focused clinical model may support a staged, sequential use of KMT relative to CBT rather than assuming concurrent delivery. Figure 2 summarizes these sequential implementation points for considering KMT as a preparatory or subsequent adjunct when patient-level barriers limit delivered CBT dose. When participation capacity is expected to be insufficient for CBT to be started and sustained, KMT can be considered **first** as a preparatory intervention, with CBT initiated when pre-specified participation-readiness indicators suggest that a minimally adequate CBT dose can be delivered. When participation difficulties are observed in a CBT attempt, or when there is a history of incomplete CBT courses, KMT can be considered after the difficulty is identified, with CBT re-offered when the identified participation barrier has improved sufficiently to attempt a minimally adequate CBT dose. In research protocols, these readiness indicators should be specified in advance and distinguished from the subsequent CBT participation endpoints used to evaluate initiation, session continuity, delivered dose, and between-session follow-through. Concurrent delivery of KMT and CBT may be considered, but it is not treated as the default clinical model here, although concurrent delivery of KMT at the initiation of CBT for bipolar remains a question that may be evaluated in future research. Table 3 specifies research design and reporting priorities for testing whether KMT reduces patient-level participation barriers that limit delivered CBT dose in bipolar disorder.

**Table 3:** Research priorities for KMT as a candidate adjunct to CBT participation in bipolar disorder.

Priority/decision point	Participation endpoints to report (psychotherapy dose)	Feasibility and acceptability indicators	Design and reporting priorities	Categories of assessment	Rationale and key sources
Engagement window: failure to initiate CBT or early discontinuation after initiation	CBT initiation rate (% referred who attend ≥1 session) Time from referral to first CBT session Early discontinuation timing (e.g., dropout before session X) Delivered dose: sessions offered, sessions attended, attendance frequency, proportion completing a pre-specified minimally adequate course Early therapeutic alliance (when participation/continuity is the target)	Patient-rated helpfulness/acceptability and reasons for non-initiation/dropout (brief structured items + optional qualitative) Medication adherence and other concurrent care adherence (if relevant to participation)	Report participation outcomes in sufficient detail to interpret feasibility/acceptability (do not rely on retention alone) Pre-specify the minimally adequate course threshold and discontinuation definitions Document CBT fidelity/competence if CBT “dose” is an endpoint (treatment integrity)	Alliance and engagement, expectancy and satisfaction, implementation acceptability and feasibility, discontinuation and attrition reasons and barriers, CBT fidelity and competence (observer rated)	Discontinuation often occurs early in structured CBT programs, truncating delivered dose, alliance is linked to outcomes and plausibly relates to continuity when participation is the endpoint (Strauss and Johnson, 2006; Docteur et al., 2018; Miklowitz et al., 2021; Chiang and Miklowitz, 2023).
Practice window: persistent failure of between-session practice despite continued treatment contact	Homework/assignment completion (rate, timeliness, quality if feasible) Adherence to self-monitoring (e.g., % days completed, missingness patterns) Completion of planned behavior-change tasks (goal attainment or task completion)	Patient-reported feasibility, burden and barriers to between-session work Optional objective/time-stamped indicators (apps, logs)	Specify and report assignment types and expected dose (what counts as “complete”) Distinguish “attendance without practice” from disengagement and dropout Consider triangulating homework with self-report, therapist ratings, and objective logs	Homework completion and quality, barriers to homework, treatment enactment and adherence, cross-EBPT, self-monitoring for bipolar course, insight and cognition as optional moderators of follow-through	Qualitative evidence suggests depressive symptoms and perceived excessive demands interfere with home practice; inaccurate self-appraisal/reduced insight may affect task calibration and follow-through (Hanssen et al., 2020; ÖZDEL et al., 2021; Grover et al., 2023; Wels et al., 2023; Yilmaz et al., 2025a).
Context window: participation constraints with comorbidity and unstable support	CBT participation endpoints above (initiation, continuity, delivered dose, practice) reported within clinically meaningful subgroups	Barriers related to social instability (housing, transportation, caregiving), and support availability (brief structured items)	Pre-specify comorbid substance use and adherence difficulties as moderators Report participation outcomes stratified by these moderators (rather than assuming uniform engagement barriers)	Substance use severity, medication adherence, service engagement, support and social needs, barriers to care	Clinician and therapist reports identify comorbid substance use, adherence difficulties, and social instability as barriers undermining continuity and follow-through (Stein et al., 2015; Veseth et al., 2017; Keramatian et al., 2023).
KMT implementation fidelity: document adherence and confirm ketosis	Align KMT measures with CBT participation endpoints to support adjunct inference (e.g., temporal coupling of KMT adherence and CBT session or practice metrics)	KMT initiation rate and time to initiation, adherence indicators (diet records, monitoring completion) Ketosis verification (measure type and frequency, proportion/time in target range) Burden/acceptability (participant ratings, adverse effects, dietetic support intensity) Mixed methods process evaluation when feasible	Report dietetic support model (structured support, individualized adjustments) and monitoring schedule Interpret adjunct effects cautiously if ketosis is not established or adherence is low Pre-specify ketosis targets and the biomarker used (blood BHB, urine ketones, breath acetone)	Ketosis confirmed, diet adherence, implementation acceptability and feasibility, burden and satisfaction	Adjunct interpretability depends on documenting adherence and verifying ketosis, bipolar KMT pilots often use dietetic support and frequent monitoring that can quantify feasibility, acceptability, and burden (Sethi and Ford, 2022; Needham et al., 2023; Rog et al., 2024; Sethi et al., 2024).
Guideline-bounded evaluation window: restrict adjunct evaluation to periods when CBT participation is a realistic target.	Identify for whom KMT is associated with improved CBT initiation, continuity, and between-session follow-through, and for whom it is not, using delivered-dose metrics and mechanisms of attrition	Define and monitor participation capacity and clinical stability (as inclusion/interpretation of prerequisite or required condition)	Align with guideline-consistent care (CBT not recommended in acute mania) pre-specify stability criteria and how phase changes affect analysis use participation outcomes as primary endpoints, symptom outcomes can be secondary/exploratory in adjunct-to-participation designs	State severity and stability as prerequisite or required condition, global functioning	CBT is not recommended in acute mania, adjunct evaluation is most appropriate when collaborative CBT work and follow-through are feasible (Yatham et al., 2018; Miklowitz et al., 2021; Chiang and Miklowitz, 2023; Keramatian et al., 2023).

This table outlines research priorities for evaluating ketogenic metabolic therapy (KMT) as a candidate adjunct to cognitive behavioral therapy (CBT) delivery in bipolar disorder using psychotherapy-defined participation endpoints. It specifies assessment targets that support quantification of delivered CBT dose, including initiation, session continuity, and between-session follow-through, and it identifies contextual participation barriers, KMT implementation indicators including ketosis verification, and timing considerations relevant to interpreting participation outcomes within guideline-consistent care. Acronyms: BD, bipolar disorder; CBT, cognitive behavioral therapy; EBPT, evidence-based psychological treatment; KMT, ketogenic metabolic therapy.

Validated instruments and standardized measurement approaches can be used to measure CBT participation processes and the factors that determine delivered dose, including therapeutic alliance, implementation acceptability and feasibility, and observer rated treatment integrity assessed through fidelity and competence methods (Muse and McManus, 2013; Weiner et al., 2017; Cameron et al., 2018; Goldberg et al., 2020). Standardized homework and between session adherence measures quantify enacted CBT dose by capturing practice completion and quality rather than attendance alone (Kazantzis et al., 2016). With these tools, the adjunct question can be tested at prespecified implementation points by assessing whether participation capacity supports CBT initiation, continuity, and between session follow through (Fernandez et al., 2015; Kazantzis et al., 2016; Weiner et al., 2017).

When KMT is implemented prior to CBT, the clinical question is whether patient-level participation capacity improves enough for CBT to begin and establish early continuity. Clinicians can assess this using early participation indicators that map directly onto CBT delivery, including transition from recommendation to initiation, early attendance continuity (Docteur et al., 2018), and initiation of routine between-session monitoring and practice (Docteur et al., 2018; ÖZDEL et al., 2021; Chiang and Miklowitz, 2023). Premature discontinuation can occur early in structured CBT programs, and early attendance patterns and discontinuation timing are clinically informative for judging whether an adjunct strategy increases the likelihood that an intended CBT dose will be delivered (Docteur et al., 2018; Miklowitz et al., 2021),

When patient-level participation difficulties are observed in a CBT attempt, KMT can be considered as a subsequent adjunctive intervention rather than concurrent delivery, with the clinical objective of improving participation capacity so that CBT can be re-offered and completed. In this scenario, clinician assessment is more informative when it specifies the observed participation problem, such as repeated missed sessions, early discontinuation risk, or persistent non-completion of monitoring and planned practice, and then assesses whether a later CBT course shows improved session continuity and between-session follow-through (Docteur et al., 2018; ÖZDEL et al., 2021). Qualitative evidence from bipolar psychotherapy programs requiring home practice indicates that low energy, reduced concentration, and perceived excessive task demands can undermine adherence and it supports a clinical approach that treats homework failure as a measurable participation barrier rather than assuming it reflects motivation alone (Hanssen et al., 2020; ÖZDEL et al., 2021).

Across staged implementations, clinical assessment is more informative when it tracks psychotherapy participation endpoints and the factors that shape engagement and continuity. Participation endpoints include CBT initiation status, session continuity, premature discontinuation, and completion of between-session monitoring and assignments (Docteur et al., 2018; ÖZDEL et al., 2021). Complementary clinical indicators include therapeutic alliance and perceived social support (Strauss and Johnson, 2006; Veseth et al., 2017), comorbid substance use and adherence difficulties that interfere with psychosocial care delivery (Stein et al., 2015), and insight and self-appraisal accuracy when these affect planning and task calibration (Grover et al., 2023; Wels et al., 2023).

When CBT is indicated but cannot be initiated, is discontinued before an adequate dose is delivered, or cannot be translated into between-session practice despite ongoing treatment contact, the adjunct question is whether KMT should be offered as a preparatory or subsequent intervention to reduce participation-limiting burden enough for CBT initiation, session continuity, and between-session follow-through to become feasible. This participation-capacity framing keeps inference narrow and guideline-consistent while the bipolar KMT evidence base remains preliminary (Docteur et al., 2018; Yatham et al., 2018; Miklowitz et al., 2021; Sethi and Ford, 2022; Chiang and Miklowitz, 2023; Keramatian et al., 2023; Rog et al., 2024).

## 6 Conclusion

CBT is an evidence-based adjunctive psychotherapy for bipolar disorder, with meta-analytic support for benefit when added to standard care (Oud et al., 2016; Chiang et al., 2017; Miklowitz et al., 2021). However, the clinical value of CBT depends on whether patients can initiate treatment, sustain session continuity, and complete between-session practice that delivers CBT's active ingredients in daily life (Docteur et al., 2018; Miklowitz et al., 2021; Chiang and Miklowitz, 2023). Patient-level participation barriers in bipolar disorder function as a clinically meaningful constraint on delivered dose and real-world benefit, particularly in routine care where psychotherapy remains underused and premature discontinuation occurs even in structured programs (Docteur et al., 2018; Chiang and Miklowitz, 2023).

Clinical implementation may be clinically appropriate when KMT is used sequentially relative to CBT, either as a preparatory intervention when participation capacity is insufficient for CBT initiation or early continuity, or as a subsequent adjunct after participation difficulties or incomplete CBT courses, with CBT re-offered when participation endpoints improve. Clinician assessment is more informative when it is anchored to observable participation outcomes and the factors that shape engagement, including alliance and social stability (Strauss and Johnson, 2006; Veseth et al., 2017), comorbid substance use and adherence difficulties (Stein et al., 2015), and insight or self-appraisal limitations when these affect planning and task calibration (Grover et al., 2023; Wels et al., 2023). Premature discontinuation can occur early in structured CBT programs, making early attendance patterns clinically informative when judging whether an adjunct strategy increases the likelihood that an intended CBT dose will be delivered (Jones et al., 2015; Docteur et al., 2018; Hautzinger and A2 BipoLife Consortium, 2024).

Given heterogeneity in bipolar disorder presentations, participation barriers, and likely response to KMT, future trials should test this participation-capacity proposition in adequately powered designs using standardized psychotherapy participation outcomes. Trials should also include careful characterization of who benefits and who does not, and qualitative analyses that clarify feasibility, burden, and discontinuation (Oud et al., 2016; Hanssen et al., 2020; Miklowitz et al., 2021). Symptom change remains an important outcome, but it is not the sole clinical rationale for CBT delivery in bipolar disorder, because CBT targets relapse prevention and functional recovery skills that remain relevant when symptoms are intermittent, residual, or already improving (Chiang et al., 2017; Yatham et al., 2018; Keramatian et al., 2023).

This conceptual analysis positions KMT as a candidate adjunct by narrowing the question to participation capacity rather than disorder-level efficacy claims. The current bipolar KMT literature remains early and does not support conclusions about comparative effectiveness, but it consistently describes change in domains that overlap with patient-level participation constraints, including mood stability, sleep, energy, anxiety, cognition, and functioning. Together, these points define a clinical conceptual framework that operationalizes KMT as a candidate adjunct using psychotherapy-defined endpoints of CBT initiation, session continuity, and between-session follow-through.
